# Age-related references in national public health, technology appraisal and clinical guidelines and guidance: documentary analysis

**DOI:** 10.1093/ageing/afw235

**Published:** 2016-12-18

**Authors:** Lynne F. Forrest, Jean Adams, Yoav Ben-Shlomo, Stefanie Buckner, Nick Payne, Melanie Rimmer, Sarah Salway, Sarah Sowden, Kate Walters, Martin White

**Affiliations:** 1 Institute of Health & Society, Newcastle University, Newcastle upon Tyne, Newcastle upon Tyne, UK; 2 School of GeoSciences, University of Edinburgh, Edinburgh, UK; 3 MRC Epidemiology Unit, University of Cambridge, Cambridge, Cambridgeshire, UK; 4 School of Social and Community Medicine, University of Bristol, Bristol, UK; 5 Institute of Public Health, University of Cambridge, Cambridge, Cambridgeshire, UK; 6 School of Health and Related Research, University of Sheffield, Sheffield, UK; 7 Centre for Ageing and Population Studies, University College London, UK

**Keywords:** older-age, NICE guidance, clinical, public health, technology appraisal

## Abstract

**Background:**

older people may be less likely to receive interventions than younger people. Age bias in national guidance may influence entire public health and health care systems. We examined how English National Institute for Health & Care Excellence (NICE) guidance and guidelines consider age.

**Methods:**

we undertook a documentary analysis of NICE public health (*n* = 33) and clinical (*n* = 114) guidelines and technology appraisals (*n* = 212). We systematically searched for age-related terms, and conducted thematic analysis of the paragraphs in which these occurred (‘age-extracts’). Quantitative analysis explored frequency of age-extracts between and within document types. Illustrative quotes were used to elaborate and explain quantitative findings.

**Results:**

2,314 age-extracts were identified within three themes: age documented as an *a-priori* consideration at scope-setting (518 age-extracts, 22.4%); documentation of differential effectiveness, cost-effectiveness or other outcomes by age (937 age-extracts, 40.5%); and documentation of age-specific recommendations (859 age-extracts, 37.1%). Public health guidelines considered age most comprehensively. There were clear examples of older-age being considered in both evidence searching and in making recommendations, suggesting that this can be achieved within current processes.

**Conclusions:**

we found inconsistencies in how age is considered in NICE guidance and guidelines. More effort may be required to ensure age is consistently considered. Future NICE committees should search for and document evidence of age-related differences in receipt of interventions. Where evidence relating to effectiveness and cost-effectiveness in older populations is available, more explicit age-related recommendations should be made. Where there is a lack of evidence, it should be stated what new research is needed.

## Introduction

Substantial research has examined inequity of access to and receipt of health care and public health interventions by age [1–3]. Older people may be less likely to receive potentially beneficial interventions, due to factors at individual, family, community and system level [1, 4–7], leading to worse outcomes [8]. Given current trends towards an ageing population [9], it is important to understand where and why age-related differences in access to and receipt of effective interventions occur and identify strategies to overcome these.

In the UK, the National Service Framework for Older People (2001) highlighted the need for ‘rooting out age-discrimination’ (p12) in Health and Social Care [10]. The UK Equality Act (2010) came into force on 1st October 2012 making age discrimination in the provision of services and public functions, including health care and public health, unlawful. Where age is considered a relevant factor in clinical and public health decision-making, the Equality Act (2010) requires ‘objective justification’ (p10) in accordance with evidence-based guidelines [11]. Elsewhere, the World Health Organization is consulting on a Global Strategy and Action Plan on Ageing and Health with a focus on equitable access to interventions [12].

One important source of information for those planning and delivering public health and health care interventions is national guidance and guidelines. In England, the National Institute for Health & Care Excellence (NICE) produces evidence-based guidance (mandatory) and guidelines (advisory) for health, public health and social care (see Box 1). NICE is internationally recognised as a role model in this area [13]. If age, or other, biases are present in guidance and guidelines, this may influence entire systems.

Box 1. **National Institute for Health & Clinical Excellence (NICE) guidance and guidelines**

**Table:** 

NICE guidelines make recommendations that are determined by independent committees on a wide range of topics, based on the best available evidence of what works, and what it costs. NICE also produces social value judgements relating to equity that committees must take into account when making recommendations. Guidance are developed using similar processes, but are mandatory. We included two types of guidelines and one type of guidance in this study:
**Public health guidelines**
These are advisory and make recommendations for populations and individuals in relation to activities, policies and strategies that can help prevent disease or improve health.
**NICE clinical guidelines**
These are recommendations on how health care professionals and others should care for people with specific conditions. Healthcare professionals are advised to take the guidelines into account when exercising clinical judgement, while making decisions appropriate to the individual circumstances and wishes of patients.
**Technology appraisals**
These provide statutory guidance on clinical needs and practice when prescribing drugs or technologies to improve health outcomes or prevent disease, and thus are mandatory.

We sought to understand whether and how age is considered in NICE guidelines and guidance.

## Methods

We systematically searched three types of NICE documents to identify all references to age and categorised these into themes. We then explored the frequency of these references to age overall and within themes, within and across document types.

### Document inclusion criteria

We included three types of documents of most relevance to health and care practitioners: clinical guidelines, public health guidelines and technology appraisals (see Box 1). NICE guidance and guidelines are produced in multiple formats and each final guideline or guidance is accompanied by a variety of supporting documents. We focused on final documents that professionals searching for these would be likely to find and use.

Documents available from the list at http://www.nice.org.uk/guidance in July 2014 were considered for inclusion. This list included full versions of public health guidelines and technology appraisals, and shortened versions of clinical guidelines. We did not include supporting documents or ‘fuller’ versions of clinical guidelines. Guidelines and guidance specific to young people, children, or pregnant women were excluded. Documents for exclusion were identified by L.F.F. and independently checked by N.P. Disagreements were resolved by discussion.

### Document searching and data extraction

A systematic electronic search of included documents was used to locate all references to ‘age’, ‘old’, ‘elder’ and related terms, e.g. ‘aged’, ‘older’ and ‘elderly’. When such age-related terms were identified, the full paragraph around each (referred to as ‘age-extracts’) was extracted verbatim for thematic analysis. This ensured that context and meaning were retained during analysis, but meant that more than one age-related term could be included per age-extract. Age-extracts were the primary unit of analysis.

### Analysis and presentation of data

Age-extracts were coded using a framework of themes and subthemes, which was inductively and iteratively developed by L.F.F. and checked by S.B. We identified the total number of age-extracts falling within each theme and subtheme, as well as the number specifically referring to older-age. A number of themes were excluded from further consideration. These were use of ‘age’ unrelated to chronological human age (e.g. the age of studies); age included in job titles (e.g. Professor of Old Age Psychiatry); references to other NICE guidance; and references to children only.

Data extraction and thematic coding were conducted by L.F.F. Five random age-extracts per theme were checked by S.B. Disagreements were resolved by discussion.

We tabulated the total number of age-extracts overall, and within each theme and subtheme, across document types. We used Poisson regression (after checking that assumptions were met) to compare the total number of age-extracts across document type, using the log number of documents as the offset. We derived the relative rate of age-extracts by exponentiating the coefficients and tested for heterogeneity by using the Wald test for document type.

The results section presents quantitative information with example quotes from age-extracts used to illustrate and help explain this.

## Results

A total of 359 documents containing 2,314 age-extracts (a mean of 6.4 age-extracts per document) were included in the analyses. Age-extracts fell into three themes: age documented as an *a-priori* consideration at scope-setting; documentation of differential effectiveness, cost-effectiveness or other outcomes by age; and documentation of age-specific recommendations.

Table 1 shows the distribution of age-extracts overall and within themes, across document types. Public health (relative rate 2.68, 95% CI 2.41 to 2.99, *P* < 0.001) and clinical guidelines (relative rate 1.14, 95% CI 1.04 to 1.25, *P* = 0.006) contained a greater number of age-extracts than technology appraisals (comparator), with strong evidence of statistical heterogeneity (*P* < 0.001).

**Table 1.** Distribution of age-extracts overall and within themes across document type

	Public health guidelines (*n* = 33)	Clinical guidelines (*n* = 114)	Technology appraisals (*n* = 212)	Total (*n* = 359)
All themes				
Age-extracts, *n*	476	699	1,139	2,314
Mean age-extracts/document, *n*	14.4	6.1	5.4	6.4
Theme 1: Age documented as an *a-priori* consideration in guidance scope				
Age-extracts, *n*	127	213	178	518
Mean age-extracts/document, *n*	3.8	1.9	0.8	1.4
Theme 2: Documentation of differential effectiveness, cost-effectiveness or other outcomes by age				
Age-extracts, *n*	193	47	697	937
Mean age-extracts/document, *n*	5.8	0.4	3.3	2.6
Theme 3: Documentation of age-specific recommendations				
Age-extracts, *n*	156	439	264	859
Mean age-extracts/document, *n*	4.7	3.9	1.2	2.4

Table 2 details the framework of included themes and subthemes, with the number of age-extracts in these by document type. Below, we discuss key findings within each theme.

**Table 2.** Age-related themes and subthemes in public health, clinical and technology appraisal guidance

Theme and subtheme	Public health (*n* = 33)		Clinical (*n* = 114)		Technology appraisal (*n* = 212)		Total (*n* = 359)	
	Documents, *n* (%)	Age-extracts, *n*	Documents, *n* (%)	Age-extracts, *n*	Documents, *n* (%)	Age-extracts, *n*	Documents, *n* (%)	Age-extracts, *n*
Theme 1: Age documented as an *a-priori* consideration in guidance scope								
Age of population guidelines aimed at	8 (24)	11	49 (43)	98	24 (11)	34	81 (23)	143
Age of population guidelines aimed at (older-age specific)	1 (3)	3	6 (5)	42	8 (4)	9	15 (4)	54
Age in guideline scope	14 (42)	17	0	0	0	0	14 (4)	17
Age in guideline scope (older-age specific)	2 (6)	3	0	0	0	0	2 (1)	3
Statistics describing problem by age	24 (73)	61	22 (19)	31	44 (21)	54	90 (26)	146
Statistics describing problem by age (older-age specific)	6 (18)	10	14 (12)	15	25 (12)	27	45 (13)	52
Age stated as risk factor for problem	10 (30)	18	39 (34)	64	56 (26)	81	105 (29)	163
Age stated as risk factor for problem (older-age specific)	8 (24)	11	29 (25)	36	50 (24)	64	87 (24)	111
Statement of why age is an important factor to consider	11 (33)	20	18 (16)	20	8 (4)	9	37 (10)	49
Statement of why age is an important factor to consider (older-age specific)	6 (18)	9	8 (7)	9	6 (3)	7	20 (6)	25
Theme 2: Documentation of differential effectiveness, cost-effectiveness or other outcomes by age								
Age as an inclusion criterion in effectiveness studies	13 (39)	42	0	0	74 (35)	151	87 (24)	193
Evidence statements of differential effectiveness by age	16 (49)	66	4 (4)	12	33 (16)	61	53 (15)	139
Evidence statements of differential effectiveness by age (older-age specific)	15 (46)	47	4 (4)	12	14 (7)	27	33 (9)	86
Limitations or gaps in evidence of effectiveness by age	13 (39)	24	17 (15)	21	24 (11)	35	54 (15)	80
Limitations or gaps in evidence of effectiveness by age (older-age specific)	4 (12)	12	12 (11)	15	9 (4)	14	25 (7)	41
Age used in cost-effectiveness models	0	0	0	0	80 (38)	167	80 (22)	167
Evidence statements of differential cost-effectiveness by age	15 (46)	32	2 (2)	2	44 (21)	144	61 (17)	178
Evidence statements of differential cost-effectiveness by age (older-age specific)	6 (18)	11	2 (2)	2	24 (11)	83	32 (9)	96
Limitations or gaps in evidence of cost-effectiveness by age	7 (21)	9	0	0	26 (12)	40	33 (9)	49
Limitations or gaps in evidence of cost-effectiveness by age (older-age specific)	2 (6)	3	0	0	6 (3)	8	8 (2)	11
Age as a reason why interventions not offered/ineffective in older people	7 (21)	19	5 (4)	5	21 (10)	34	33 (9)	58
Age of those included in trials different to those at risk	1 (3)	1	0	0	33 (16)	49	34 (10)	50
Adverse effects in older people	0	0	6 (5)	7	13 (6)	16	19 (5)	23
Theme 3: Documentation of age-specific recommendations								
Age taken into consideration when making recommendations	22 (67)	39	0	0	82 (39)	220	104 (29)	259
Equality Act taken into consideration when making recommendations	1 (3)	1	0	0	10 (5)	16	11 (3)	17
Target population for recommendations age specific	7 (21)	15	0	0	0	0	7 (2)	15
Target population for recommendations (older-age specific)	3 (9)	9	0	0	0	0	3 (1)	9
Priority for implementation of recommendations age specific	0	0	31 (27)	47	0	0	31 (9)	47
Priority for implementation of recommendations (older-age specific)	0	0	17 (15)	22	0	0	17 (5)	22
Further effectiveness research recommended by age	22 (67)	34	31 (27)	60	4 (2)	4	57 (16)	98
Further effectiveness research recommended by age (older-age specific)	4 (12)	11	19 (17)	45	2 (1)	2	25 (7)	58
Further cost-effectiveness research recommended by age	13 (39)	21	6 (5)	7	0	0	19 (5)	28
Further cost-effectiveness research recommended by age (older-age specific)	4 (12)	10	3 (3)	4	0	0	7 (2)	14
Other age-related recommendations	18 (55)	47	73 (64)	325	14 (7)	40	105 (29)	412
Other age-related recommendations (older-age specific)	8 (24)	17	48 (42)	149	8 (4)	28	64 (18)	194

### Theme 1: age documented as an *a-priori* consideration in guidance scope

Almost half of clinical guidelines (*n* = 49, 43%) and a quarter of public health guidelines (*n* = 8, 24%) and technology appraisals (*n* = 81, 23%) were aimed at age-specific groups. Very few of any type of document were aimed at older-age groups specifically (*n* = 15, 4%).

Only public health guidelines documented considering age at scope-setting, although this was done in less than half of cases (*n* = 14, 42%). On 11 occasions, the same question was listed: ‘Does the effectiveness of the intervention vary with different characteristics within the target population, such as age?’. This question was not documented in any technology appraisals or clinical guidelines.

Public health guidelines (*n* = 25, 76%) were more likely than clinical guidelines (*n* = 22, 19%) or technology appraisals (*n* = 44, 21%) to report statistics describing the problem addressed by age. Similarly, public health guidelines were more likely to describe why age might be an important factor to consider than other documents. However, this was only done in one-third (*n* = 11, 33%) of public health guidelines. Around one-third of all documents identified age as a risk factor for the problem addressed (*n* = 105, 29%), particularly older-age (*n* = 87, 24%).

Box 2 provides illustrative quotations from age-extracts coded within theme 1.

Box 2. **Illustrative quotations from age-extracts within theme 1**

**Table:** 

**Age in guideline scope**
‘Are interventions tailored to sub-sets of the smoking population (for example, pregnant women, older smokers) more effective with them than generic interventions?’ *PH1 Brief interventions and referral for smoking cessation*
‘How does the effectiveness vary with age, gender, class, ethnicity, etc.?’ *PH2 Four commonly used methods to increase physical activity*
‘What are the most effective and cost-effective ways for primary and residential care services to promote the mental wellbeing of older people?’ *PH16 Occupational therapy and physical activity interventions to promote the mental wellbeing of older people in primary care and residential care*
**Statistics describing problem considered by age**
‘53% of men aged 16–24 achieved the recommended activity levels, compared with 8% of men aged 75 and over. Among women, 29–31% aged 16–54 reached the recommended level. However, the same was only true of 3% of women aged 75 and over.’ *PH2 Four commonly used methods to increase physical activity*
**Age stated as risk factor for problem considered**
‘In people between the ages of 45 and 49 years, the incidence is about 20 per 100,000. In those aged 75 and older, the annual incidence is over 300 cases per 100,000 men and over 200 cases per 100,000 women.’ *TA105 Laparoscopic surgery for colorectal cancer*
**Statements of why age is an important factor to consider**
‘More than 250,000 older people (aged 66 and older) living in England in private households reported experiencing maltreatment from a family member.’ *PH50 Domestic violence and abuse – how services can respond effectively*

### Theme 2: documentation of differential effectiveness, cost-effectiveness or other outcomes by age

Detailed considerations of evidence of effectiveness and cost-effectiveness were not included in the clinical guideline documents included. While no clinical guidelines described age-related evidence limitations on cost-effectiveness, 17 (15%) described age-related limitations in evidence of effectiveness. These primarily related specifically to older-age (*n* = 12, 11%).

Overall, public health guidelines were at least twice as likely as technology appraisals to report evidence of differential effectiveness and cost-effectiveness by age, as well as age-related evidence gaps in evidence of effectiveness and cost-effectiveness. While the majority of evidence statements of differential effectiveness by age specifically related to older-age, this was not the case for cost-effectiveness evidence statements and age-related evidence gaps.

A number of evidence statements in public health guidelines were based on qualitative work relating to understanding of risk, and of the relevance, appropriateness or acceptability of an intervention for older populations. Some public health guidelines also referred to factors that might account for lower uptake or effectiveness of interventions in older populations, such as frailty, poorer health, age-related differences in perception of relative risk, use of tools designed for younger people and scarcity of resources.

A number of technology appraisals (*n* = 33, 16%) and public health guidelines (*n* = 1, 3%) noted that the age profile of those included in relevant trials was generally younger to the age profile of those at risk of the problem studied.

Illustrative quotes from age-extracts in theme 2 are shown in Box 3.

Box 3. **Illustrative quotations from age-extracts within theme 2**

**Table:** 

**Evidence statements of differential effectiveness/cost-effectiveness by age**
‘One…study reports a reduction in effectiveness in promoting CVD [cardio-vascular disease] awareness in older participants. Two…studies report no differences in effectiveness according to age.’ *PH25 Prevention of cardiovascular disease*
‘A meta-analysis of exercise capacity with dual-chamber pacing compared with single-chamber ventricular pacing demonstrated no difference…for patients older than 75 years…but there was…for patients younger than 75.’ *TA88 Dual-chamber pacemakers for symptomatic bradycardia due to sick sinus syndrome and or atrioventricular block*
**Limitations or gaps in evidence of effectiveness/cost-effectiveness by age**
‘there are…no studies specifically addressing people aged 75 and over.’ *CG180 The management of atrial fibrillation*
‘No evidence was found of effective or cost-effective interventions to promote mental wellbeing in older people living in residential care.’ *PH16 Mental wellbeing and older people*
**Age as a reason why interventions not offered/ineffective in older people**
‘One qualitative study…found that age was widely perceived to influence access to services…Focus groups revealed that staff appeared to have knowledge of the benefits for older people but that scarcity of resources prevented them offering more accessible and appropriate services.’ *PH15 Identifying and supporting people most at risk of dying prematurely*
‘The PDG [programme development group] considered that people over age 74…might benefit from type 2 diabetes risk assessment and prevention…However, it recognised that many of the risk-assessment tools are not validated for this age group.’ *PH38 Preventing type 2 diabetes – risk identification and interventions for individuals at high risk*
**Age of those included in trials different to that those at risk**
‘The Committee…noted that the mean age of patients in the trial was 56 years but…the average age of men with gout in UK practice was around 10 years older.’ *TA291 Gout (tophaceous, severe debilitating, chronic) – pegloticase*

### Theme 3: documentation of age-specific recommendations

Two-thirds of public health guidelines (*n* = 22, 67%), no clinical guidelines and two-fifths of technology appraisals (*n* = 82, 39%) documented that they took age into account when making recommendations. Many of these recommendations focused on ensuring people were not excluded from interventions on the basis of age alone.

Throughout, there were many documented discussions of whether age should be taken into account in recommendations and, in documents published since 2012, whether recommendations should be revised in light of the Equalities Act (2010). No such revisions were recommended and documents often concluded that intervention decisions should be made on an individual basis.

Evidence of effectiveness and cost-effectiveness of new technologies are often supplied by manufacturers to NICE technology appraisal panels. Panels appear to spend substantial time testing and discussing this evidence. Age is regularly examined during this process (*n* = 82, 39%), but mostly in terms of how it is used in cost-effectiveness models.

Only public health guidelines made age-specific practice recommendations, but only in one-fifth of cases (*n* = 7, 21%). Less than half of these recommendations were specific to older-age (*n* = 3, 9%). In contrast, only clinical guidelines prioritised practice recommendations according to age, but these were only present in one-quarter (*n* = 31, 27%). Around half were older-age specific (*n* = 17, 15%).

Age-specific research recommendations were present across guideline types—but were least often found in technology appraisals. Two-thirds (*n* = 22, 67%) of public health guidelines included recommendations for further age-specific effectiveness evidence and more than one-third (*n* = 13, 39%) for further age-specific cost-effectiveness evidence. Comparable figures for clinical guidelines were 31 (27%) and 6 (5%); and for technology appraisals were 4 (2%) and 0. In most cases, less than half of these were older-age specific.

Box 4 shows illustrative quotations from age-extracts in theme 3.

Box 4. **Illustrative quotations from age-extracts within theme 3**

**Table:** 

**Age taken into consideration when making recommendations**
‘a small population of older patients who are not fit enough to receive chemotherapy may not have access to an alternative treatment and so may be disadvantaged. The Committee agreed that this was not an issue of age discrimination because other factors can also affect whether people are fit enough to receive chemotherapy, such as comorbidities.’ *TA257 Breast cancer (metastatic hormone receptor) – lapatinib and trastuzumab (with aromatase inhibitor)*
‘The Committee considered whether NICE's duties under the equalities legislation required it to alter or to add to its recommendations…the Committee concluded that…there was no need to alter or add to its recommendations.’ *TA282 Idiopathic pulmonary fibrosis – pirfenidone*
**Target population for recommendations age specific**
‘All COPD patients still smoking, regardless of age, should be encouraged to stop, and offered help to do so, at every opportunity.’ *CG101 Chronic obstructive pulmonary disease*
‘Offer people aged 80 years and over the same antihypertensive drug treatment as people aged 55–80 years, taking into account any comorbidities.’ *CG127 Hypertension*
‘Patient-specific factors (including age…) should not be barriers to referral for joint surgery.’ *CG177 Osteoarthritis*
**Further effectiveness/cost-effectiveness research recommended by age**
‘How does effectiveness and cost effectiveness vary for…people aged 75 and over?’ *PH32 Type 2 diabetes*
‘Future studies should be sufficiently powered to detect changes in mental wellbeing…In addition, the outcome measures used should be appropriate to detect change across different groups of older people.’ *PH16 Mental wellbeing and older people*

## Discussion

### Summary of findings

This is the first attempt we are aware of to analyse how age in general, and older-age in particular, is considered in national health and health care guidelines and guidance. Using NICE as a case study, we found 2,314 age-extracts across 359 public health and clinical guidelines and technology appraisals. These fell into three broad themes: age documented as an *a-priori* consideration at scope-setting; documentation of differential effectiveness, cost-effectiveness or other outcomes by age; and documentation of age-specific recommendations.

Age was not considered consistently across the three document types. In general, public health guidelines considered age more comprehensively, but this was still not consistent across all public health guidelines.

A lack of explicit age-related recommendations within guidelines and guidance could result in uncertainties for practitioners and commissioners. In the absence of evidence, guideline developers might choose to highlight the need for further research. While many public health guidelines did this, few technology appraisals and clinical guidelines did so.

### Study limitations

NICE produces a range of different guidelines and guidance. We focused on clinical and public health guidelines and technology appraisals as we considered these to be most relevant for practitioners and commissioners. Although multiple documents related to each guideline and piece of guidance are available, we focused on the most front-facing of these that practitioners would be most likely to find. This maximised the relevance of our findings. However, our findings are not necessarily generalisable to other types of NICE document, or to other countries.

Documentary analysis is labour intensive and time-consuming [14]. We searched and extracted data from hundreds of large documents. It was not feasible to read all of these in full. Instead we automated searching for extracts, but coded these by hand. Duplicate thematic coding helped increase the validity of this.

It would have been interesting to formally test our qualitative assessment that the Equalities Act influenced how age was considered. However, small numbers in many cells in Table 2 meant this was not considered appropriate.

There was inconsistency in the information included in the different types of documents studied. In particular, the clinical guideline documents included did not incorporate the evidence used to make recommendations (although this is available elsewhere), while public health guidelines and technology appraisal documents did. This may explain some of the differences between document types found. Reporting variations may also reflect variations in approach.

Using documentary analysis meant we only included information that had been documented. Committees developing guidance and guidelines may have considered many issues that were not explicitly recorded in included documents.

We searched specifically for age-related terms. Instances when age was indirectly referred to, for example in terms of life expectancy or frailty, may have been excluded, underestimating the frequency of age-extracts. However, there is little reason to believe this would vary systematically across document types and so constitute bias.

We excluded guidance and guidelines with a specific focus on children, young people and pregnant women. We did not make judgements about which included documents should have considered age. However, it could be argued that all should have done—even if just to state that guidance and guidelines were relevant across the age spectrum.

It is unclear how much impact NICE guidance has on either front-line practice or what services are prioritised for funding at a local or national level and thus how much the differences identified here might influence practice [15]. However, it has been suggested that increasing the specificity of guidelines is likely to improve implementation [16].

### Interpretation of results

Since the implementation of the UK Equality Act (2010) in 2012, which made age discrimination in the provision of services and public functions unlawful, NICE is legally obliged to ensure that age discrimination does not occur. While the Act does appear to have resulted in age being more explicitly considered in technology appraisals, this often appeared to involve a post-hoc check for compliance, rather than an integration of the Act's principles throughout the development process. It is possible that further developments to embed the principles of the Equality Act (2010) in NICE processes are planned, but we are not aware of anything in particular.

Legal obligations are only one aspect of equity. The concept of ‘embedded inequity’ proposes that consideration should also be made of whether omissions in methodological process, outcome measures, and individual context and circumstances might lead to discrimination [17]. As others have reported [18], we found that public health guidelines appear to more consistently (but not universally) include consideration of age than other included documents. This may reflect the use of a conceptual framework to inform public health guidance development and explicitly consider inequalities since at least 2009. While some NICE processes help ensure embedded equity, others do not.

Nearly half of public health guidelines and one-fifth of technology appraisals reported evidence of differential effectiveness and cost-effectiveness by age. Interventions may be less cost-effective in older people due to their shorter life expectancy [19]. However, in many instances where prevalence increases with age, numbers needed to treat will be smaller in older people and this may increase the cost-effectiveness of some interventions. Furthermore, the benefits of interventions to older people, in terms of reduced morbidity, improved quality of life and maintained independence, are likely to be different, but no less valuable, than those to younger people [20]. More consideration of how age-related differences in benefit can be taken into account in cost-effectiveness calculations is required as well as consideration of alternative methodological approaches that may be more suited for older people [21].

### Comparison with previous findings

Others have found, in different contexts, a lack of clear age-related clinical recommendations [22] and suggested that NICE make explicit reference to age where there is evidence of age-related inequities in receipt of interventions [22]. However, little is known about whether, and where, age-related inequities in receipt of health care and public health interventions occur. More concerted and systematic action is required to identify age-related differences in receipt interventions to guide development of guidelines and guidance.

### Implications for research, policy and practice

A lack of evidence on effectiveness and cost-effectiveness in older populations was often noted in included documents. Some might argue that it is unwise to provide interventions without evidence of effectiveness or cost-effectiveness. This is likely to result in age-related differences in receipt of interventions. Others may feel that older people should have access to all interventions until such time as evidence emerges of a lack of effectiveness or cost-effectiveness. While the Equality Act (2010) would favour the latter approach, over-provision of ineffective interventions to older people may result in iatrogenesis and waste. Research on the effectiveness and cost-effectiveness of interventions in older populations is required to overcome any ambiguity in who should be offered interventions. Until this is available, clear guidance on how practitioners should act in the face of absence of evidence is required.

Many included documents identified, as previously [21, 23], that the age profile of those included in trials and evaluations was not representative of the population most at risk. There is little scientific justification for this. Future research should focus on providing evidence relevant to populations most at risk.

In several instances, documents avoided the issue of chronological age by referring to biological age, frailty or ‘fitness’, leaving decisions on assessment of these to practitioners. Variability in how practitioners make such judgements may be due to unconscious prejudices [24]. Better understanding is required of how the risks and benefits of interventions are evaluated by practitioners and older people in the absence of explicit guidance, and what contributions these assessments may make to inequalities in receipt of interventions.

## Conclusions

We found inconsistencies in how age is considered in NICE public health and clinical guidelines and technology appraisals. There were some clear examples of older-age being considered in both searching for evidence and in making specific recommendations, suggesting that this can be achieved within current processes.

NICE deserves credit for openly discussing equity issues in decision-making [25]. More effort may be required to ensure age is consistently considered across all processes. Future NICE guidance should systematically search for and document evidence of age-related differences in receipt of interventions. Where evidence is available relating to effectiveness and cost-effectiveness in older populations, more explicit age-related recommendations should be made. Where there is a lack of evidence, guidance should formally state what new research is needed.

**Key points**

- Age bias in national guidance may influence entire public health and health care systems.
- Documentation of how age and older-age were considered in English national guidance and guidelines was not consistent.
- There were some clear examples of older-age being considered in both evidence searching and in making recommendations.
- More effort may be required to ensure age is consistently considered across all processes.

## Supplementary data

Supplementary data mentioned in the text are available to subscribers in *Age and Ageing* online.

## Authors’ contributions

Authors contributions are detailed in Appendix 1, Supplementary data are available in *Age and Ageing* online.

## Conflicts of interest

None declared.

## Funding

This work was supported by the National Institute for Health Research's School for Public Health Research (NIHR SPHR http://sphr.nihr.ac.uk/). J.A. & M.W. are members of the Centre for Diet and Activity Research (CEDAR) a UKCRC Public Health Research Centre of Excellence. The funders had no role in study design, data collection and analysis, decision to publish or preparation of the manuscript. A full funding statement is provided in Appendix 1, Supplementary data are available in *Age and Ageing* online.

## Supplementary Material

Supplementary Data

## Declarations

### Abbreviations

NICE: National Institute of Health & Clinical Excellence.

### Availability of data and materials

All data used in this manuscript are publically available from http://www.nice.org.uk/guidance
