# Supplementary material for: Age-related references in national public health, technology appraisal and clinical guidelines and guidance: documentary analysis
Source: Age Ageing. 2016 Dec 18;46(3):500–8. doi: 10.1093/ageing/afw235 (PMC5405753; doi:10.1093/ageing/afw235)
Supplement: Supplementary Data [file aa-16-0448-File001.docx]

## **Supplementary Data**

## For Age and Ageing paper: *Age-related references in national public health, technology appraisal and clinical guidelines and guidance: documentary analysis*

## **Appendix 1. Age in NICE Guidance**

**Funding**

This work was supported by the National Institute for Health Research's School for Public Health Research (NIHR SPHR http://sphr.nihr.ac.uk/). The views expressed are those of the authors and not necessarily those of the NHS, the NIHR or the Department of Health. SPHR is funded by the NIHR. SPHR is a partnership between the Universities of Sheffield, Bristol, Cambridge; UCL; The London School for Hygiene and Tropical Medicine; The Peninsula College of Medicine and Dentistry; the LiLaC collaboration between the Universities of Liverpool and Lancaster and Fuse; The Centre for Translational Research in Public Health, a collaboration between Newcastle, Durham, Northumbria, Sunderland and Teesside Universities. JA & MW are members of the Centre for Diet and Activity Research (CEDAR) a UKCRC Public Health Research Centre of Excellence - funding from the British Heart Foundation, Cancer Research UK, Economic and Social Research Council, Medical Research Council, the National Institute for Health Research, and the Wellcome Trust, under the auspices of the UK Clinical Research Collaboration, is gratefully acknowledged. The funders had no role in study design, data collection and analysis, decision to publish, or preparation of the manuscript.
